# 2-Amino-4-(4-chloro­phen­yl)-5,6-dihydro­benzo[*h*]quinoline-3-carbonitrile–3-amino-1-(4-chloro­phen­yl)-9,10-dihydro­phenanthrene-2,4-dicarbonitrile (1/4)

**DOI:** 10.1107/S1600536811040529

**Published:** 2011-10-08

**Authors:** Abdullah M. Asiri, Abdulrahman O. Al-Youbi, Hassan M. Faidallah, Seik Weng Ng

**Affiliations:** a Chemistry Department, Faculty of Science, King Abdulaziz University, PO Box 80203 Jeddah, Saudi Arabia; b Center of Excellence for Advanced Materials Research, King Abdulaziz University, PO Box 80203 Jeddah, Saudi Arabia; c Department of Chemistry, University of Malaya, 50603 Kuala Lumpur, Malaysia

## Abstract

The asymmetric unit of the 1:4 title co-crystal of 2-amino-4-(4-chloro­phen­yl)-5,6-dihydro­benzo[*h*]quinoline-3-carbonitrile and 3-amino-1-(4-chloro­phen­yl)-9,10-dihydro­phenanthrene-2,4-dicarbonitrile, 0.2C_20_H_14_ClN_3_·0.8C_22_H_14_ClN_3_, has the atoms of the fused-ring system and those of the amino, cyano and chloro­phenyl substitutents overlapped. The fused-ring system is buckled owing to the ethyl­ene linkage in the central ring. There are two independent overlapped mol­ecules in the asymmetric unit. In one independent mol­ecule, the two flanking aromatic rings are twisted by 24.4 (1)° and the ring of the chloro­phenyl substituent is twisted by 87.3 (1)° relative to the amino- and cyano-bearing aromatic ring. In the second mol­ecule, the respective dihedral angles are 26.1 (1) and 57.8 (1)°. The two independent mol­ecules are linked by N—H⋯N hydrogen bonds into dimers.

## Related literature

For similar co-crystals, see: Asiri *et al.* (2011*a* ▶,*b* ▶).
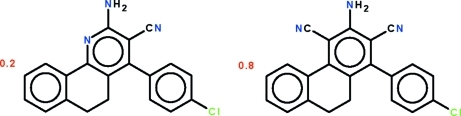


## Experimental

### 

#### Crystal data

0.2C_20_H_14_ClN_3_·0.8C_22_H_14_ClN_3_
*M* *_r_* = 351.01
Monoclinic, 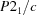

*a* = 19.2576 (7) Å
*b* = 9.5103 (2) Å
*c* = 20.2266 (7) Å
β = 114.018 (4)°
*V* = 3383.7 (2) Å^3^
*Z* = 8
Cu *K*α radiation
μ = 2.06 mm^−1^
*T* = 100 K
0.30 × 0.20 × 0.10 mm

#### Data collection

Agilent SuperNova Dual diffractometer with an Atlas detector
Absorption correction: multi-scan (*CrysAlis PRO*; Agilent, 2010 ▶) *T*_min_ = 0.577, *T*_max_ = 0.821
12442 measured reflections
6686 independent reflections
6272 reflections with *I* > 2σ(*I*)
*R*_int_ = 0.032

#### Refinement

*R*[*F* ^2^ > 2σ(*F* ^2^)] = 0.051
*wR*(*F* ^2^) = 0.130
*S* = 1.05
6686 reflections
471 parameters
H-atom parameters constrained
Δρ_max_ = 0.44 e Å^−3^
Δρ_min_ = −0.65 e Å^−3^

### 

Data collection: *CrysAlis PRO* (Agilent, 2010 ▶); cell refinement: *CrysAlis PRO*; data reduction: *CrysAlis PRO*; program(s) used to solve structure: *SHELXS97* (Sheldrick, 2008 ▶); program(s) used to refine structure: *SHELXL97* (Sheldrick, 2008 ▶); molecular graphics: *X-SEED* (Barbour, 2001 ▶); software used to prepare material for publication: *publCIF* (Westrip, 2010 ▶).

## Supplementary Material

Crystal structure: contains datablock(s) global, I. DOI: 10.1107/S1600536811040529/zs2147sup1.cif

Structure factors: contains datablock(s) I. DOI: 10.1107/S1600536811040529/zs2147Isup2.hkl

Supplementary material file. DOI: 10.1107/S1600536811040529/zs2147Isup3.cml

Additional supplementary materials:  crystallographic information; 3D view; checkCIF report

## Figures and Tables

**Table 1:** Hydrogen-bond geometry (Å, °)

*D*—H⋯*A*	*D*—H	H⋯*A*	*D*⋯*A*	*D*—H⋯*A*
N2—H21⋯N4	0.88	2.14	2.931 (3)	149
N5—H52⋯N3	0.88	2.33	3.136 (3)	152

**Fig. 1.**
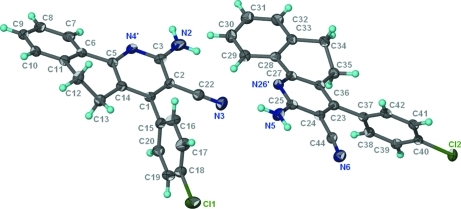
Thermal ellipsoid plot (Barbour, 2001) of C20H14N3Cl at the 70% probability level. Hydrogen atoms are drawn as spheres of arbitrary radius.

**Fig. 2.**
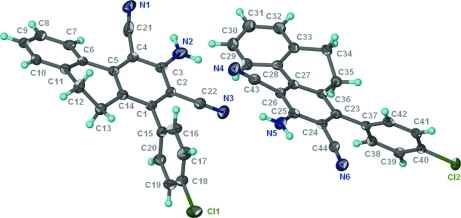
Thermal ellipsoid plot (Barbour, 2001) of C22H14N3Cl at the 70% probability level. Hydrogen atoms are drawn as spheres of arbitrary radius.

## supplementary crystallographic information

### Comment

2-Amino-5,6-dihydro-4-phenyl-benzoquinoline-3-carbonitrile is synthesized from the reaction of the α-substituted cinnamonitrile, C_6_H_5_CH═C(CN)_2_, with α-tetralone in a reaction that is catalyzed by ammonium acetate. The synthesis when conducted under microwave irradiation leads to an improved yield. In previous studies, we obtained instead di-carbonitrile substituted dihydrophenanthrenes (3-amino-1-(4-methoxyphenyl)-9,10-dihydrophenanthrene-2,4-dicarbonitrile and 3-amino-1-(2*H*-1,3-benzodioxol-5-yl)- 9,10-dihydrophenanthrene-2,4-dicarbonitrile) with 4-methoxybenzaldehyde and piperonaldehyde in syntheses that differed slightly from the reported ones as we used substituted benzaldehydes, α-tetralone and ethyl cyanoacetate along with a molar excess of ammonium acetate.

In this study, the reaction of 4-chlorobenzaldehyde, α-tetralone and ethyl cyanoacetate yielded the co-crystal of the title compound 2-amino-4-(4-chlorophenyl)-5,6-dihydrobenzoquinoline-3-carbonitrile (C_20_H_14_N_3_Cl) and 3-amino-1-(4-chlorophenyl)-9,10-dihydrophenanthrene-2,4-dicarbonitrile (C_22_H_14_N_3_Cl) with the components present in a 1: 4 molar ratio (Scheme I). The fused-ring system is buckled owing to the ethylene linkage in the central ring, the two flanking aromatic rings being twisted by 24.4 (1)°. Relative to the amino- and cyano-bearing aromatic ring, the benzene ring is twisted by 87.3 (1)° in one independent overlapped molecule. For the second molecule, the respective dihedral angles are 26.1 (1) ° and 57.8 (1) ° (Fig. 1 and Fig. 2). Two molecules are linked by an N—H···N hydrogen bonds (Table 1) to generate dimers.

### Experimental

A mixture of 4-chlorobenzaldehyde (1.41 g,10 mmol), α-tetralone (1.46 g, 10 mmol), ethyl cyanoacetate (1.13 g, 10 mmol) and ammonium acetate (6.16 g, 80 mmol) in absolute ethanol (50 ml) was refluxed for 6 h. The mixture was allowed to cool and the precipitate that formed was filtered, washed with water, dried and recrystallized from DMF.

### Refinement

Carbon-bound H-atoms were placed in calculated positions [C—H = 0.95–0.99, N—H = 0.88 Å; *U*_iso_(H) 1.2*U*_eq_(C,*N*)] and were included in the refinement in the riding model approximation. The compound is a co-crystal of 2-amino-4-(4-chlorophenyl)-5,6-dihydrobenzoquinoline-3-carbonitrile (C_20_H_14_N_3_Cl) and 3-amino-1-(4-chlorophenyl)-9,10-dihydrophenanthrene-2,4-dicarbonitrile (C_22_H_14_N_3_Cl). The first component is a dihydrobenzoquinoline and has only one amino substituent. The second component is a dihydrophenanthrene with two amino substituents. The two-coordinate N atom of one component molecule occupies the same site as the three-coordinate C atom of the second overlapped molecule. The asymmetric unit consists of two independent overlapped molecules. For one, the occupancy refined to nearly 0.33 and for the other, to nearly 0.07. The occupancies were then fixed at these ratios.

### Crystal data

**Table:**

0.2C_20_H_14_ClN_3_·0.8C_22_H_14_ClN_3_	*F*(000) = 1452.8
*M**_r_* = 351.01	*D*_x_ = 1.378 Mg m^−^^3^
Monoclinic, *P*2_1_/*c*	Cu *K*α radiation, λ = 1.54184 Å
Hall symbol: -P 2ybc	Cell parameters from 9520 reflections
*a* = 19.2576 (7) Å	θ = 2.5–74.4°
*b* = 9.5103 (2) Å	µ = 2.06 mm^−^^1^
*c* = 20.2266 (7) Å	*T* = 100 K
β = 114.018 (4)°	Prism, brown-orange
*V* = 3383.7 (2) Å^3^	0.30 × 0.20 × 0.10 mm
*Z* = 8	

### Data collection

**Table:**

Agilent SuperNova Dual diffractometer with an Atlas detector	6686 independent reflections
Radiation source: SuperNova (Cu) X-ray Source	6272 reflections with *I* > 2σ(*I*)
Mirror	*R*_int_ = 0.032
Detector resolution: 10.4041 pixels mm^-1^	θ_max_ = 74.6°, θ_min_ = 2.5°
ω scans	*h* = −23→19
Absorption correction: multi-scan (*CrysAlis PRO*; Agilent, 2010)	*k* = −11→11
*T*_min_ = 0.577, *T*_max_ = 0.821	*l* = −22→25
12442 measured reflections	

### Refinement

**Table:**

Refinement on *F*^2^	Primary atom site location: structure-invariant direct methods
Least-squares matrix: full	Secondary atom site location: difference Fourier map
*R*[*F*^2^ > 2σ(*F*^2^)] = 0.051	Hydrogen site location: inferred from neighbouring sites
*wR*(*F*^2^) = 0.130	H-atom parameters constrained
*S* = 1.05	*w* = 1/[σ^2^(*F*_o_^2^) + (0.0541*P*)^2^ + 4.4722*P*] where *P* = (*F*_o_^2^ + 2*F*_c_^2^)/3
6686 reflections	(Δ/σ)_max_ = 0.001
471 parameters	Δρ_max_ = 0.44 e Å^−^^3^
0 restraints	Δρ_min_ = −0.65 e Å^−^^3^

### Fractional atomic coordinates and isotropic or equivalent isotropic displacement parameters (Å^2^)

**Table:**

	*x*	*y*	*z*	*U*_iso_*/*U*_eq_	Occ. (<1)
Cl1	0.47649 (4)	0.54771 (7)	0.91711 (3)	0.03619 (16)	
Cl2	1.19971 (3)	0.62336 (6)	0.76577 (3)	0.02717 (14)	
N2	0.41277 (11)	0.3060 (2)	0.46275 (10)	0.0303 (5)	
H22	0.3945	0.2761	0.4177	0.036*	
H21	0.4590	0.3410	0.4827	0.036*	
N3	0.53363 (11)	0.4429 (2)	0.63456 (11)	0.0309 (5)	
N5	0.70451 (10)	0.4628 (2)	0.65759 (10)	0.0242 (4)	
H52	0.6564	0.4376	0.6390	0.029*	
H51	0.7232	0.5107	0.6983	0.029*	
N6	0.87451 (11)	0.5819 (2)	0.77852 (10)	0.0249 (4)	
C1	0.34901 (12)	0.3426 (2)	0.61038 (11)	0.0185 (4)	
C2	0.39635 (11)	0.3465 (2)	0.57321 (11)	0.0188 (4)	
C3	0.36991 (12)	0.2981 (2)	0.50123 (11)	0.0213 (4)	
C5	0.25137 (11)	0.2336 (2)	0.50655 (11)	0.0173 (4)	
C6	0.17630 (11)	0.1630 (2)	0.47299 (11)	0.0175 (4)	
C7	0.15869 (12)	0.0681 (2)	0.41558 (11)	0.0219 (4)	
H7	0.1960	0.0458	0.3977	0.026*	
C8	0.08727 (13)	0.0061 (2)	0.38444 (11)	0.0231 (4)	
H8	0.0755	−0.0565	0.3448	0.028*	
C9	0.03309 (12)	0.0358 (2)	0.41145 (11)	0.0215 (4)	
H9	−0.0160	−0.0057	0.3900	0.026*	
C10	0.05081 (12)	0.1262 (2)	0.46977 (11)	0.0200 (4)	
H10	0.0139	0.1447	0.4887	0.024*	
C11	0.12166 (12)	0.1903 (2)	0.50104 (11)	0.0185 (4)	
C12	0.14005 (12)	0.2922 (2)	0.56290 (12)	0.0229 (4)	
H12A	0.1067	0.2733	0.5884	0.027*	
H12B	0.1302	0.3894	0.5438	0.027*	
C13	0.22293 (12)	0.2787 (2)	0.61594 (11)	0.0225 (4)	
H13A	0.2355	0.3544	0.6526	0.027*	
H13B	0.2307	0.1873	0.6414	0.027*	
C14	0.27531 (12)	0.2883 (2)	0.57713 (11)	0.0192 (4)	
C15	0.38013 (12)	0.3938 (2)	0.68678 (11)	0.0199 (4)	
C16	0.36942 (14)	0.5320 (3)	0.70204 (12)	0.0281 (5)	
H16	0.3417	0.5943	0.6636	0.034*	
C17	0.39890 (14)	0.5809 (3)	0.77331 (13)	0.0290 (5)	
H17	0.3911	0.6754	0.7839	0.035*	
C18	0.43959 (13)	0.4887 (3)	0.82785 (11)	0.0247 (5)	
C19	0.45232 (13)	0.3507 (3)	0.81436 (12)	0.0260 (5)	
H19	0.4810	0.2894	0.8529	0.031*	
C20	0.42214 (13)	0.3036 (2)	0.74306 (12)	0.0236 (4)	
H20	0.4303	0.2091	0.7327	0.028*	
C22	0.47249 (12)	0.4012 (2)	0.60785 (11)	0.0229 (4)	
C23	0.87585 (11)	0.4285 (2)	0.61845 (11)	0.0175 (4)	
C24	0.82793 (12)	0.4631 (2)	0.65336 (11)	0.0183 (4)	
C25	0.75014 (12)	0.4275 (2)	0.62329 (11)	0.0190 (4)	
C27	0.77056 (12)	0.3061 (2)	0.52568 (11)	0.0184 (4)	
C28	0.74168 (11)	0.2139 (2)	0.46097 (11)	0.0185 (4)	
C29	0.68059 (13)	0.1213 (2)	0.44618 (12)	0.0233 (4)	
H29	0.6585	0.1112	0.4802	0.028*	
C30	0.65195 (13)	0.0441 (2)	0.38272 (13)	0.0264 (5)	
H30	0.6097	−0.0166	0.3729	0.032*	
C31	0.68490 (14)	0.0557 (3)	0.33362 (13)	0.0276 (5)	
H31	0.6638	0.0062	0.2890	0.033*	
C32	0.74870 (13)	0.1394 (2)	0.34957 (12)	0.0244 (5)	
H32	0.7727	0.1431	0.3168	0.029*	
C33	0.77796 (12)	0.2181 (2)	0.41305 (11)	0.0197 (4)	
C34	0.84650 (12)	0.3108 (2)	0.43101 (11)	0.0217 (4)	
H34A	0.8298	0.4075	0.4137	0.026*	
H34B	0.8776	0.2755	0.4058	0.026*	
C35	0.89469 (12)	0.3136 (2)	0.51267 (11)	0.0202 (4)	
H35A	0.9183	0.2203	0.5286	0.024*	
H35B	0.9360	0.3836	0.5236	0.024*	
C36	0.84698 (12)	0.3508 (2)	0.55402 (11)	0.0178 (4)	
C37	0.95655 (11)	0.4762 (2)	0.65269 (10)	0.0171 (4)	
C38	1.01649 (12)	0.3796 (2)	0.67461 (11)	0.0190 (4)	
H38	1.0058	0.2822	0.6658	0.023*	
C39	1.09139 (12)	0.4242 (2)	0.70914 (11)	0.0194 (4)	
H39	1.1319	0.3582	0.7243	0.023*	
C40	1.10595 (12)	0.5666 (2)	0.72103 (11)	0.0197 (4)	
C41	1.04796 (12)	0.6654 (2)	0.69953 (11)	0.0206 (4)	
H41	1.0592	0.7627	0.7078	0.025*	
C42	0.97326 (12)	0.6197 (2)	0.66573 (11)	0.0201 (4)	
H42	0.9330	0.6863	0.6513	0.024*	
C44	0.85661 (11)	0.5307 (2)	0.72266 (11)	0.0196 (4)	
N1	0.26563 (17)	0.2046 (3)	0.32991 (15)	0.0287 (6)	0.67
N4	0.57603 (11)	0.3302 (2)	0.49932 (11)	0.0265 (4)	0.93
C4	0.29686 (11)	0.2425 (2)	0.46910 (10)	0.0181 (4)	0.67
C21	0.2743 (2)	0.2161 (4)	0.3913 (2)	0.0298 (8)	0.67
C26	0.72231 (11)	0.3513 (2)	0.55831 (11)	0.0186 (4)	0.93
C43	0.64133 (13)	0.3348 (2)	0.52387 (12)	0.0205 (5)	0.93
N4'	0.29686 (11)	0.2425 (2)	0.46910 (10)	0.0181 (4)	0.33
N26'	0.72231 (11)	0.3513 (2)	0.55831 (11)	0.0186 (4)	0.07

### Atomic displacement parameters (Å^2^)

**Table:**

	*U*^11^	*U*^22^	*U*^33^	*U*^12^	*U*^13^	*U*^23^
Cl1	0.0500 (4)	0.0405 (3)	0.0163 (3)	−0.0087 (3)	0.0117 (2)	−0.0076 (2)
Cl2	0.0201 (2)	0.0315 (3)	0.0268 (3)	−0.0075 (2)	0.0064 (2)	0.0028 (2)
N2	0.0256 (10)	0.0490 (13)	0.0178 (9)	0.0029 (9)	0.0103 (8)	−0.0021 (9)
N3	0.0229 (10)	0.0450 (13)	0.0250 (10)	−0.0089 (9)	0.0099 (8)	−0.0051 (9)
N5	0.0183 (9)	0.0361 (11)	0.0202 (9)	−0.0021 (8)	0.0097 (7)	−0.0072 (8)
N6	0.0239 (9)	0.0276 (10)	0.0226 (10)	−0.0002 (8)	0.0088 (8)	−0.0034 (8)
C1	0.0183 (10)	0.0193 (10)	0.0160 (9)	0.0014 (8)	0.0050 (8)	0.0006 (8)
C2	0.0164 (9)	0.0202 (10)	0.0170 (10)	−0.0019 (8)	0.0038 (8)	−0.0013 (8)
C3	0.0209 (10)	0.0239 (11)	0.0172 (10)	0.0028 (8)	0.0058 (8)	−0.0002 (8)
C5	0.0179 (9)	0.0168 (10)	0.0169 (9)	0.0022 (7)	0.0068 (8)	0.0019 (7)
C6	0.0176 (9)	0.0185 (10)	0.0156 (9)	0.0004 (8)	0.0059 (8)	0.0029 (8)
C7	0.0244 (10)	0.0267 (11)	0.0158 (9)	0.0011 (9)	0.0093 (8)	−0.0003 (8)
C8	0.0278 (11)	0.0230 (11)	0.0162 (10)	−0.0042 (9)	0.0067 (8)	−0.0023 (8)
C9	0.0213 (10)	0.0220 (10)	0.0184 (10)	−0.0041 (8)	0.0051 (8)	0.0019 (8)
C10	0.0189 (10)	0.0220 (10)	0.0202 (10)	−0.0015 (8)	0.0090 (8)	0.0023 (8)
C11	0.0195 (10)	0.0182 (10)	0.0181 (10)	0.0000 (8)	0.0077 (8)	0.0001 (8)
C12	0.0191 (10)	0.0258 (11)	0.0254 (11)	−0.0016 (8)	0.0107 (9)	−0.0085 (9)
C13	0.0219 (10)	0.0282 (11)	0.0189 (10)	−0.0028 (9)	0.0098 (8)	−0.0043 (8)
C14	0.0189 (10)	0.0219 (10)	0.0169 (9)	0.0005 (8)	0.0073 (8)	0.0013 (8)
C15	0.0165 (9)	0.0267 (11)	0.0164 (9)	−0.0033 (8)	0.0067 (8)	−0.0026 (8)
C16	0.0311 (12)	0.0282 (12)	0.0201 (11)	0.0059 (9)	0.0054 (9)	0.0002 (9)
C17	0.0344 (12)	0.0249 (12)	0.0253 (11)	0.0020 (10)	0.0097 (10)	−0.0068 (9)
C18	0.0272 (11)	0.0333 (12)	0.0145 (10)	−0.0063 (9)	0.0094 (8)	−0.0056 (9)
C19	0.0300 (12)	0.0289 (12)	0.0174 (10)	−0.0014 (9)	0.0080 (9)	0.0028 (9)
C20	0.0264 (11)	0.0236 (11)	0.0201 (10)	−0.0005 (9)	0.0089 (9)	−0.0009 (8)
C22	0.0223 (11)	0.0287 (11)	0.0190 (10)	−0.0021 (9)	0.0096 (9)	−0.0024 (8)
C23	0.0178 (10)	0.0164 (10)	0.0174 (9)	0.0020 (7)	0.0063 (8)	0.0031 (8)
C24	0.0193 (10)	0.0193 (10)	0.0156 (9)	0.0009 (8)	0.0065 (8)	0.0008 (8)
C25	0.0190 (10)	0.0210 (10)	0.0178 (9)	0.0027 (8)	0.0083 (8)	0.0028 (8)
C27	0.0205 (10)	0.0174 (10)	0.0176 (9)	0.0017 (8)	0.0079 (8)	0.0039 (8)
C28	0.0181 (9)	0.0181 (10)	0.0179 (9)	0.0024 (8)	0.0058 (8)	0.0034 (8)
C29	0.0233 (10)	0.0235 (11)	0.0237 (11)	−0.0017 (8)	0.0100 (9)	0.0009 (9)
C30	0.0261 (11)	0.0223 (11)	0.0285 (11)	−0.0052 (9)	0.0087 (9)	−0.0027 (9)
C31	0.0334 (12)	0.0235 (11)	0.0230 (11)	−0.0029 (9)	0.0086 (9)	−0.0050 (9)
C32	0.0306 (11)	0.0226 (11)	0.0218 (10)	−0.0021 (9)	0.0125 (9)	−0.0024 (8)
C33	0.0223 (10)	0.0164 (10)	0.0198 (10)	0.0007 (8)	0.0081 (8)	0.0025 (8)
C34	0.0256 (11)	0.0225 (11)	0.0193 (10)	−0.0043 (8)	0.0114 (9)	0.0002 (8)
C35	0.0205 (10)	0.0222 (10)	0.0199 (10)	−0.0001 (8)	0.0103 (8)	0.0009 (8)
C36	0.0188 (10)	0.0184 (10)	0.0168 (9)	0.0016 (8)	0.0080 (8)	0.0028 (8)
C37	0.0178 (10)	0.0203 (10)	0.0140 (9)	−0.0010 (8)	0.0074 (8)	0.0002 (7)
C38	0.0210 (10)	0.0186 (10)	0.0187 (10)	−0.0004 (8)	0.0093 (8)	0.0006 (8)
C39	0.0176 (9)	0.0241 (11)	0.0175 (9)	0.0023 (8)	0.0082 (8)	0.0007 (8)
C40	0.0174 (9)	0.0268 (11)	0.0151 (9)	−0.0059 (8)	0.0066 (8)	0.0011 (8)
C41	0.0249 (10)	0.0187 (10)	0.0185 (10)	−0.0035 (8)	0.0092 (8)	0.0016 (8)
C42	0.0222 (10)	0.0204 (10)	0.0186 (10)	0.0017 (8)	0.0092 (8)	0.0031 (8)
C44	0.0163 (9)	0.0205 (10)	0.0220 (10)	0.0015 (8)	0.0077 (8)	0.0013 (8)
N1	0.0317 (15)	0.0271 (15)	0.0198 (14)	−0.0052 (12)	0.0027 (12)	−0.0082 (11)
N4	0.0202 (10)	0.0335 (12)	0.0266 (10)	−0.0015 (8)	0.0102 (8)	−0.0057 (9)
C4	0.0170 (9)	0.0197 (9)	0.0160 (9)	0.0020 (7)	0.0052 (7)	−0.0002 (7)
C21	0.0244 (17)	0.0307 (19)	0.034 (2)	−0.0111 (14)	0.0118 (15)	−0.0104 (15)
C26	0.0175 (9)	0.0204 (10)	0.0183 (9)	0.0001 (8)	0.0076 (8)	0.0008 (8)
C43	0.0246 (12)	0.0213 (11)	0.0175 (10)	0.0004 (9)	0.0105 (9)	−0.0031 (8)
N4'	0.0170 (9)	0.0197 (9)	0.0160 (9)	0.0020 (7)	0.0052 (7)	−0.0002 (7)
N26'	0.0175 (9)	0.0204 (10)	0.0183 (9)	0.0001 (8)	0.0076 (8)	0.0008 (8)

### Geometric parameters (Å, °)

**Table:**

Cl1—C18	1.742 (2)	C19—C20	1.392 (3)
Cl2—C40	1.744 (2)	C19—H19	0.9500
N2—C3	1.348 (3)	C20—H20	0.9500
N2—H22	0.8800	C23—C36	1.401 (3)
N2—H21	0.8800	C23—C24	1.410 (3)
N3—C22	1.148 (3)	C23—C37	1.491 (3)
N5—C25	1.364 (3)	C24—C25	1.410 (3)
N5—H52	0.8800	C24—C44	1.433 (3)
N5—H51	0.8800	C25—C26	1.403 (3)
N6—C44	1.147 (3)	C27—C26	1.408 (3)
C1—C14	1.399 (3)	C27—C36	1.410 (3)
C1—C2	1.399 (3)	C27—C28	1.483 (3)
C1—C15	1.493 (3)	C28—C29	1.402 (3)
C2—C3	1.410 (3)	C28—C33	1.407 (3)
C2—C22	1.441 (3)	C29—C30	1.383 (3)
C3—C4	1.392 (3)	C29—H29	0.9500
C5—C4	1.374 (3)	C30—C31	1.383 (3)
C5—C14	1.409 (3)	C30—H30	0.9500
C5—C6	1.484 (3)	C31—C32	1.388 (3)
C6—C7	1.399 (3)	C31—H31	0.9500
C6—C11	1.408 (3)	C32—C33	1.392 (3)
C7—C8	1.390 (3)	C32—H32	0.9500
C7—H7	0.9500	C33—C34	1.504 (3)
C8—C9	1.390 (3)	C34—C35	1.530 (3)
C8—H8	0.9500	C34—H34A	0.9900
C9—C10	1.385 (3)	C34—H34B	0.9900
C9—H9	0.9500	C35—C36	1.515 (3)
C10—C11	1.390 (3)	C35—H35A	0.9900
C10—H10	0.9500	C35—H35B	0.9900
C11—C12	1.506 (3)	C37—C38	1.399 (3)
C12—C13	1.524 (3)	C37—C42	1.402 (3)
C12—H12A	0.9900	C38—C39	1.389 (3)
C12—H12B	0.9900	C38—H38	0.9500
C13—C14	1.512 (3)	C39—C40	1.384 (3)
C13—H13A	0.9900	C39—H39	0.9500
C13—H13B	0.9900	C40—C41	1.387 (3)
C15—C16	1.385 (3)	C41—C42	1.388 (3)
C15—C20	1.392 (3)	C41—H41	0.9500
C16—C17	1.396 (3)	C42—H42	0.9500
C16—H16	0.9500	N1—C21	1.188 (5)
C17—C18	1.377 (3)	N4—C43	1.150 (3)
C17—H17	0.9500	C4—C21	1.474 (4)
C18—C19	1.383 (3)	C26—C43	1.434 (3)
			
C3—N2—H22	120.0	C36—C23—C37	122.42 (18)
C3—N2—H21	120.0	C24—C23—C37	117.86 (18)
H22—N2—H21	120.0	C23—C24—C25	121.68 (19)
C25—N5—H52	120.0	C23—C24—C44	121.61 (19)
C25—N5—H51	120.0	C25—C24—C44	116.65 (18)
H52—N5—H51	120.0	N5—C25—C26	121.59 (19)
C14—C1—C2	120.48 (19)	N5—C25—C24	120.86 (19)
C14—C1—C15	121.29 (18)	C26—C25—C24	117.52 (18)
C2—C1—C15	118.21 (18)	C26—C27—C36	119.75 (19)
C1—C2—C3	120.40 (19)	C26—C27—C28	120.81 (19)
C1—C2—C22	120.36 (19)	C36—C27—C28	119.44 (18)
C3—C2—C22	119.24 (19)	C29—C28—C33	118.7 (2)
N2—C3—C4	119.46 (19)	C29—C28—C27	122.74 (19)
N2—C3—C2	122.0 (2)	C33—C28—C27	118.57 (19)
C4—C3—C2	118.55 (19)	C30—C29—C28	121.0 (2)
C4—C5—C14	121.11 (19)	C30—C29—H29	119.5
C4—C5—C6	119.78 (18)	C28—C29—H29	119.5
C14—C5—C6	119.11 (18)	C31—C30—C29	119.9 (2)
C7—C6—C11	119.01 (19)	C31—C30—H30	120.1
C7—C6—C5	122.22 (19)	C29—C30—H30	120.1
C11—C6—C5	118.75 (18)	C30—C31—C32	120.0 (2)
C8—C7—C6	120.7 (2)	C30—C31—H31	120.0
C8—C7—H7	119.6	C32—C31—H31	120.0
C6—C7—H7	119.6	C31—C32—C33	120.7 (2)
C7—C8—C9	119.9 (2)	C31—C32—H32	119.6
C7—C8—H8	120.0	C33—C32—H32	119.6
C9—C8—H8	120.0	C32—C33—C28	119.5 (2)
C10—C9—C8	119.8 (2)	C32—C33—C34	121.61 (19)
C10—C9—H9	120.1	C28—C33—C34	118.90 (19)
C8—C9—H9	120.1	C33—C34—C35	110.99 (17)
C9—C10—C11	121.05 (19)	C33—C34—H34A	109.4
C9—C10—H10	119.5	C35—C34—H34A	109.4
C11—C10—H10	119.5	C33—C34—H34B	109.4
C10—C11—C6	119.47 (19)	C35—C34—H34B	109.4
C10—C11—C12	121.04 (18)	H34A—C34—H34B	108.0
C6—C11—C12	119.46 (18)	C36—C35—C34	111.25 (17)
C11—C12—C13	110.83 (18)	C36—C35—H35A	109.4
C11—C12—H12A	109.5	C34—C35—H35A	109.4
C13—C12—H12A	109.5	C36—C35—H35B	109.4
C11—C12—H12B	109.5	C34—C35—H35B	109.4
C13—C12—H12B	109.5	H35A—C35—H35B	108.0
H12A—C12—H12B	108.1	C23—C36—C27	119.37 (18)
C14—C13—C12	111.00 (17)	C23—C36—C35	122.44 (18)
C14—C13—H13A	109.4	C27—C36—C35	118.19 (18)
C12—C13—H13A	109.4	C38—C37—C42	118.88 (19)
C14—C13—H13B	109.4	C38—C37—C23	121.14 (19)
C12—C13—H13B	109.4	C42—C37—C23	119.94 (19)
H13A—C13—H13B	108.0	C39—C38—C37	120.9 (2)
C1—C14—C5	118.23 (18)	C39—C38—H38	119.6
C1—C14—C13	122.24 (18)	C37—C38—H38	119.6
C5—C14—C13	119.42 (18)	C40—C39—C38	118.8 (2)
C16—C15—C20	119.5 (2)	C40—C39—H39	120.6
C16—C15—C1	120.5 (2)	C38—C39—H39	120.6
C20—C15—C1	120.0 (2)	C39—C40—C41	121.9 (2)
C15—C16—C17	120.7 (2)	C39—C40—Cl2	119.12 (17)
C15—C16—H16	119.6	C41—C40—Cl2	119.01 (17)
C17—C16—H16	119.6	C40—C41—C42	118.9 (2)
C18—C17—C16	118.4 (2)	C40—C41—H41	120.5
C18—C17—H17	120.8	C42—C41—H41	120.5
C16—C17—H17	120.8	C41—C42—C37	120.7 (2)
C17—C18—C19	122.3 (2)	C41—C42—H42	119.7
C17—C18—Cl1	118.97 (19)	C37—C42—H42	119.7
C19—C18—Cl1	118.69 (18)	N6—C44—C24	174.8 (2)
C18—C19—C20	118.5 (2)	C5—C4—C3	121.06 (18)
C18—C19—H19	120.7	C5—C4—C21	127.0 (2)
C20—C19—H19	120.7	C3—C4—C21	111.5 (2)
C15—C20—C19	120.5 (2)	N1—C21—C4	170.5 (3)
C15—C20—H20	119.8	C25—C26—C27	121.57 (19)
C19—C20—H20	119.8	C25—C26—C43	115.91 (18)
N3—C22—C2	178.6 (2)	C27—C26—C43	122.23 (19)
C36—C23—C24	119.72 (19)	N4—C43—C26	174.8 (2)
			
C14—C1—C2—C3	1.1 (3)	C26—C27—C28—C29	−27.3 (3)
C15—C1—C2—C3	179.4 (2)	C36—C27—C28—C29	153.8 (2)
C14—C1—C2—C22	−179.4 (2)	C26—C27—C28—C33	153.8 (2)
C15—C1—C2—C22	−1.2 (3)	C36—C27—C28—C33	−25.2 (3)
C1—C2—C3—N2	177.0 (2)	C33—C28—C29—C30	−5.6 (3)
C22—C2—C3—N2	−2.4 (3)	C27—C28—C29—C30	175.5 (2)
C1—C2—C3—C4	−2.0 (3)	C28—C29—C30—C31	1.5 (4)
C22—C2—C3—C4	178.5 (2)	C29—C30—C31—C32	3.0 (4)
C4—C5—C6—C7	20.2 (3)	C30—C31—C32—C33	−3.3 (4)
C14—C5—C6—C7	−158.7 (2)	C31—C32—C33—C28	−0.9 (3)
C4—C5—C6—C11	−161.23 (19)	C31—C32—C33—C34	−179.3 (2)
C14—C5—C6—C11	19.9 (3)	C29—C28—C33—C32	5.2 (3)
C11—C6—C7—C8	2.8 (3)	C27—C28—C33—C32	−175.78 (19)
C5—C6—C7—C8	−178.7 (2)	C29—C28—C33—C34	−176.3 (2)
C6—C7—C8—C9	−1.5 (3)	C27—C28—C33—C34	2.7 (3)
C7—C8—C9—C10	−0.6 (3)	C32—C33—C34—C35	−145.8 (2)
C8—C9—C10—C11	1.3 (3)	C28—C33—C34—C35	35.8 (3)
C9—C10—C11—C6	0.0 (3)	C33—C34—C35—C36	−52.5 (2)
C9—C10—C11—C12	178.0 (2)	C24—C23—C36—C27	−1.1 (3)
C7—C6—C11—C10	−2.1 (3)	C37—C23—C36—C27	178.39 (19)
C5—C6—C11—C10	179.36 (19)	C24—C23—C36—C35	178.23 (19)
C7—C6—C11—C12	179.9 (2)	C37—C23—C36—C35	−2.2 (3)
C5—C6—C11—C12	1.3 (3)	C26—C27—C36—C23	6.1 (3)
C10—C11—C12—C13	144.7 (2)	C28—C27—C36—C23	−174.91 (18)
C6—C11—C12—C13	−37.3 (3)	C26—C27—C36—C35	−173.26 (19)
C11—C12—C13—C14	51.9 (3)	C28—C27—C36—C35	5.7 (3)
C2—C1—C14—C5	2.2 (3)	C34—C35—C36—C23	−146.4 (2)
C15—C1—C14—C5	−175.98 (19)	C34—C35—C36—C27	33.0 (3)
C2—C1—C14—C13	178.4 (2)	C36—C23—C37—C38	−59.7 (3)
C15—C1—C14—C13	0.2 (3)	C24—C23—C37—C38	119.9 (2)
C4—C5—C14—C1	−4.8 (3)	C36—C23—C37—C42	122.6 (2)
C6—C5—C14—C1	174.13 (19)	C24—C23—C37—C42	−57.8 (3)
C4—C5—C14—C13	178.92 (19)	C42—C37—C38—C39	0.4 (3)
C6—C5—C14—C13	−2.2 (3)	C23—C37—C38—C39	−177.34 (18)
C12—C13—C14—C1	150.0 (2)	C37—C38—C39—C40	−0.5 (3)
C12—C13—C14—C5	−33.9 (3)	C38—C39—C40—C41	0.1 (3)
C14—C1—C15—C16	−88.5 (3)	C38—C39—C40—Cl2	178.87 (15)
C2—C1—C15—C16	93.3 (3)	C39—C40—C41—C42	0.6 (3)
C14—C1—C15—C20	93.4 (3)	Cl2—C40—C41—C42	−178.23 (15)
C2—C1—C15—C20	−84.8 (3)	C40—C41—C42—C37	−0.8 (3)
C20—C15—C16—C17	−1.4 (4)	C38—C37—C42—C41	0.3 (3)
C1—C15—C16—C17	−179.5 (2)	C23—C37—C42—C41	178.03 (18)
C15—C16—C17—C18	0.8 (4)	C14—C5—C4—C3	4.0 (3)
C16—C17—C18—C19	0.3 (4)	C6—C5—C4—C3	−174.93 (19)
C16—C17—C18—Cl1	−179.78 (19)	C14—C5—C4—C21	−167.1 (3)
C17—C18—C19—C20	−0.7 (4)	C6—C5—C4—C21	14.1 (4)
Cl1—C18—C19—C20	179.38 (17)	N2—C3—C4—C5	−179.6 (2)
C16—C15—C20—C19	1.0 (3)	C2—C3—C4—C5	−0.5 (3)
C1—C15—C20—C19	179.1 (2)	N2—C3—C4—C21	−7.3 (3)
C18—C19—C20—C15	0.0 (3)	C2—C3—C4—C21	171.8 (2)
C36—C23—C24—C25	−3.2 (3)	N5—C25—C26—C27	−175.3 (2)
C37—C23—C24—C25	177.26 (19)	C24—C25—C26—C27	2.8 (3)
C36—C23—C24—C44	174.06 (19)	N5—C25—C26—C43	10.7 (3)
C37—C23—C24—C44	−5.5 (3)	C24—C25—C26—C43	−171.22 (19)
C23—C24—C25—N5	−179.5 (2)	C36—C27—C26—C25	−7.1 (3)
C44—C24—C25—N5	3.1 (3)	C28—C27—C26—C25	174.00 (19)
C23—C24—C25—C26	2.4 (3)	C36—C27—C26—C43	166.5 (2)
C44—C24—C25—C26	−175.01 (19)	C28—C27—C26—C43	−12.4 (3)

### Hydrogen-bond geometry (Å, °)

**Table:**

*D*—H···*A*	*D*—H	H···*A*	*D*···*A*	*D*—H···*A*
N2—H21···N4	0.88	2.14	2.931 (3)	149
N5—H52···N3	0.88	2.33	3.136 (3)	152
